# Macaque Monkeys Perceive the Flash Lag Illusion

**DOI:** 10.1371/journal.pone.0058788

**Published:** 2013-03-19

**Authors:** Manivannan Subramaniyan, Alexander S. Ecker, Philipp Berens, Andreas S. Tolias

**Affiliations:** 1 Department of Neuroscience, Baylor College of Medicine, Houston, Texas, United States of America; 2 Werner Reichardt Centre for Integrative Neuroscience and Institute of Theoretical Physics, University of Tübingen, Tübingen, Germany; 3 Bernstein Center for Computational Neuroscience, Tübingen, Germany; 4 Max Planck Institute for Biological Cybernetics, Tübingen, Germany; 5 Michael E. DeBakey Veterans Affairs Medical Center, Houston, Texas, United States of America; 6 Department of Computational and Applied Mathematics, Rice University, Houston, Texas, United States of America; Aalto University, School of Science, Finland

## Abstract

Transmission of neural signals in the brain takes time due to the slow biological mechanisms that mediate it. During such delays, the position of moving objects can change substantially. The brain could use statistical regularities in the natural world to compensate neural delays and represent moving stimuli closer to real time. This possibility has been explored in the context of the flash lag illusion, where a briefly flashed stimulus in alignment with a moving one appears to lag behind the moving stimulus. Despite numerous psychophysical studies, the neural mechanisms underlying the flash lag illusion remain poorly understood, partly because it has never been studied electrophysiologically in behaving animals. Macaques are a prime model for such studies, but it is unknown if they perceive the illusion. By training monkeys to report their percepts unbiased by reward, we show that they indeed perceive the illusion qualitatively similar to humans. Importantly, the magnitude of the illusion is smaller in monkeys than in humans, but it increases linearly with the speed of the moving stimulus in both species. These results provide further evidence for the similarity of sensory information processing in macaques and humans and pave the way for detailed neurophysiological investigations of the flash lag illusion in behaving macaques.

## Introduction

Neural delays arising from synaptic transmission and axonal conduction are a natural consequence of the architecture of the brain. Taking these delays into account is a fundamental step in information processing in the nervous system. How these delays affect sensory perception and whether they are compensated has fascinated both philosophers and scientists for centuries[1]. Visual signals, for example, can take more than 100 ms to reach higher order cortical areas in primates – raising the question of whether we perceive the world in real time or whether our visual perception is outdated. For instance, if a car approaches you at 70 km/h, it would actually be two meters closer than you perceive it if the brain did not compensate for delays. Although the adverse effects of such delays could be overcome at the sensorimotor level [2], [3], it is intensely debated whether afferent delays are compensated in the perceptual systems [1], [4].

**Figure 1:**
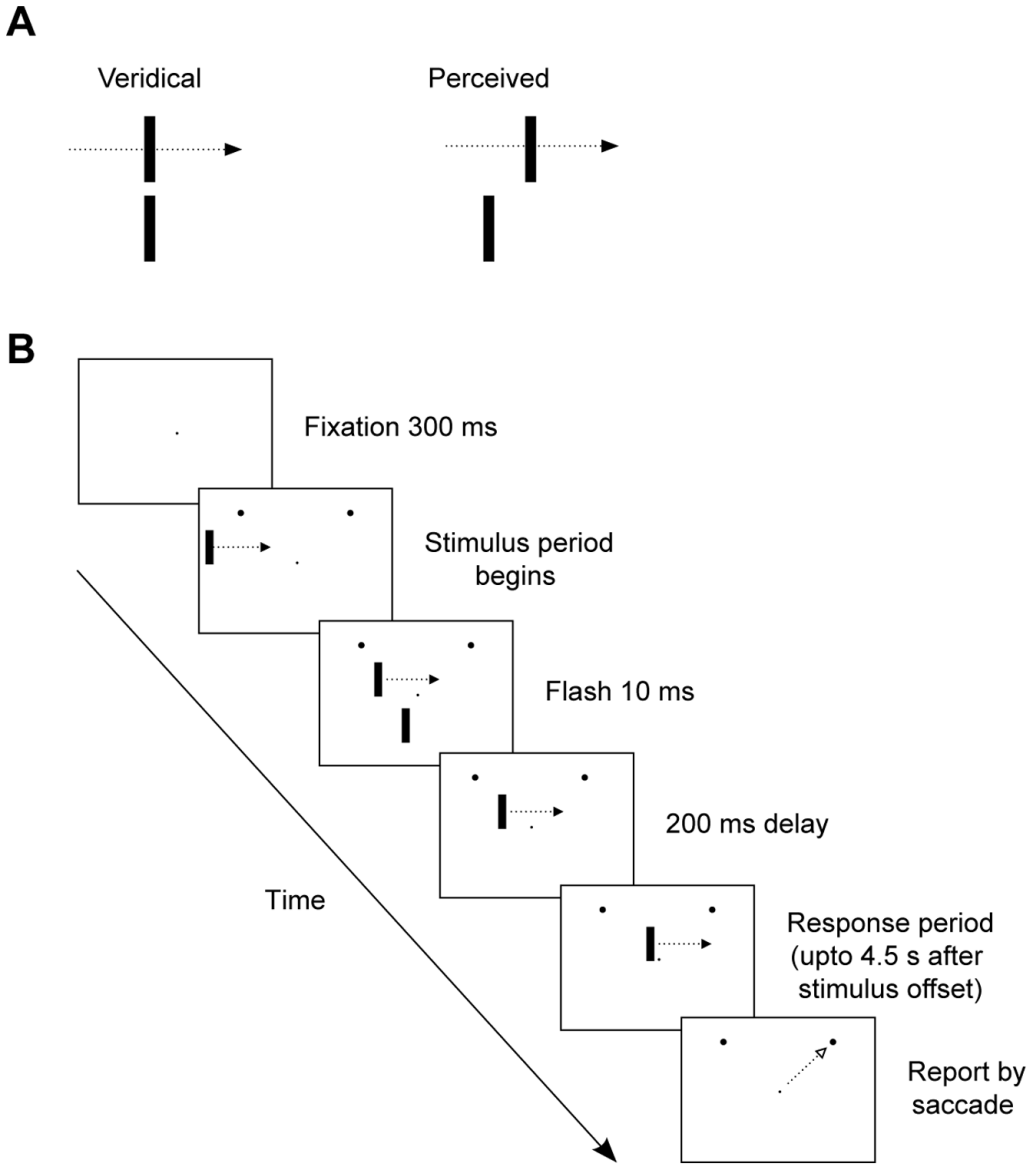
Flash lag illusion and monkey behavioral task. (A) Illustration of the flash lag illusion. A flash that is presented in vertical alignment with a moving bar appears to lag behind the moving bar. (B) The schematic shows stimuli (not drawn to scale) and events in a trial. Stimuli were presented on CRT monitors with a gray background. Subjects fixated for 300 ms after which the stimulus period began. A bright moving bar started to move at a constant speed (10, 14 or 20 °/s) from the left or right end of the monitor display. After the onset of the moving bar, another bar of the same size and luminance was flashed below the moving bar for one video frame (10 ms) at a fixed location. The flash onset time was adjusted to provide different horizontal spatial offsets. For each trial a single spatial offset was chosen randomly from a range of offsets. After a delay of 200 ms from the flash onset, subjects were allowed to report if the flash was located on the right or left side of the moving bar, using a joystick or by saccading to visual targets (illustrated as two black dots in the upper visual field). The next trial began after 1200 ms of inter-trial period.

An experimental paradigm in which neural delays and position computation of moving objects have been addressed is the flash lag illusion, where a briefly presented stimulus appears to spatially lag behind a moving stimulus at the instant when both are physically aligned (Figure 1A). Following the original discovery in 1958 [5] and its rediscovery in 1994 [6] a number of theories have been put forward to explain the illusion. Most of these fall into one of three broad categories: spatial extrapolation [6], differential latency [7]–[9] and temporal integration [10], [11]. In the spatial extrapolation model the perceptual system uses motion signals to extrapolate the moving object's current position into its future position to compensate for neural delays. This extrapolation leads to the apparent displacement of the moving stimulus relative to the flashed one since such an extrapolation does not occur for the flash, which is not predictable. The differential latency account, in contrast, posits that moving stimuli are processed faster than flashes. Hence, the neuronal representation of the flash temporally coincides with that of the moving bar located further along the motion path. Finally, according to the temporal integration models, the position of a moving object is computed as a spatial average of the positions in a time window around or after the perceived onset of the flash. Other mechanisms that have been put forward to explain the illusion include visual persistence [12], attention shifts [13] priming and backward masking [14], flash-triggered sampling of motion signals [15] and perceptual facilitation [16].

A number of psychophysical studies have addressed the flash lag illusion and have provided evidence both in favor of and against each of these accounts [1], [4], [7]–[11], [13], [15], [17]–[21]. This could suggest that the flash lag effect is likely not caused by a single physiological mechanism but rather a combination of mechanisms [22] involving many levels of the information processing hierarchy and/or a new unknown neural mechanism. To uncover the neurophysiological mechanisms of the illusion it is crucial to analyze response properties of neurons in various brain areas possibly involved in the task. A few studies have examined neural responses in the retina and early visual areas to static and moving bars with reference to the flash lag illusion [23]–[25]. These studies were conducted either in vitro [23] or in animals that were anesthetized [24] or passively fixating [25]. Although these studies have provided important clues to the underlying neural mechanisms, future studies that combine behavior and physiology in the same animal can provide additional insights into the neural mechanisms. Before using animals in such studies, it is necessary to first establish that they actually perceive the illusion – this is currently unknown.

Given how similar the macaque's visual system is to that of humans and their ability to learn complex behavioral tasks, we reasoned that they might be an ideal model system to study the flash lag illusion – if they actually perceive it. We therefore trained monkeys to report their percept when viewing flash lag stimuli. Our results show that monkeys indeed perceive the flash lag illusion and that many of its characteristics are similar in monkeys and humans, paving the way for a combined behavioral and neurophysiological investigation of the illusion in macaque monkeys.

## Results

### Monkey psychophysics

We presented two bright vertical bars on a gray background, one of which moved horizontally with uniform speed while the other one briefly appeared below it (Figure 1A). When the moving and flashed bars are presented in perfect vertical alignment, human observers typically report that at the instant they perceive the flash, the moving bar is located ahead of it (Figure 1A). We asked if monkeys also perceive the flash as lagging behind the moving bar. We trained two monkeys to report their percept by making an eye movement or moving a lever.

Training monkeys to report if they perceive an illusion is delicate: they learn their task by getting reward for correct responses, but in illusions the correct or incorrect response is not always well defined since the percept may differ from physical reality. We developed a reward scheme that allowed us to train the monkeys to report their percepts unbiased by reward. To achieve this, we initially presented moving and flashed bars at large spatial offsets (magnitude >2.5°, ‘suprathreshold trials’) and trained monkeys to indicate whether the flashed bar is left or right of the moving bar (Figure 1B). During testing, nine different spatial offsets in the range of ±2.5° were presented. In the trials with small spatial offsets (within ±1.5°, ‘subthreshold trials’), where the monkeys' percept may not be in agreement with the physical reality, no particular response was reinforced by reward; in trials with spatial offset magnitude greater than 1.5°, reward was given for reporting the veridical stimulus configuration. For further details on the reward scheme refer to the Methods and Table 1.

**Table 1:** Behavioral training and reward scheme for monkeys.

Training phase II												
Trial number	1	2	3	4	5	6	7	8	9	10	11	12
Stimulus type	S	S	S	S	S	S	S	S	S	S	S	S
Correct response?	✓		✓	✓	✓	✗	✗	✓	✗	✓	✗	✓
Rewarded?	✓		✓		✓			✓				✓
Reward block size	1	1		2		1			2			

Training phase III												
Trial number	1	2	3	4	5	6	7	8	9	10	11	12
Stimulus type	S	P	S	S	S	S	S	P	S	S	S	S
Correct response?	✓	U	✓	✓	✓	✗	✓	U	✗	✓	✓	✓
Rewarded?	✓		✓		✓		✓			✓	✓	✓
Reward block size	1	2		2		1		2			1	1

✓-Yes, P - Probe or subthreshold stimuli

✗ - No, S - Suprathreshold stimuli

U - Undefined

Monkeys were trained in three phases to report their percepts unbiased by reward. The spatial offsets used depended on the training phase. Spatial offsets with magnitude more than 1.5° are marked as ‘S’ (Suprathreshold) and offsets in the range within ±1.5° are marked as ‘P’ (Probe or subthreshold stimulus). In phase-I (not depicted here), only suprathreshold stimuli were used and every correct trial was rewarded. In training phase-II, the monkeys performed the same task as in phase-I, but without immediate reward for some trials, by requiring them to complete one or two correct trials (one-trial and two-trial reward blocks respectively) to receive reward. In the two-trial reward blocks, the monkeys received reward after completing two (not necessarily consecutive) correct trials. In training phase-III, the monkeys performed the same task as in phase-II except that the suprathreshold and subthreshold stimuli were randomly interleaved in the non-rewarded trial slot of the two-trial reward blocks. We counted the monkeys' response (labeled as U) to probe stimuli as correct. However this did not give any response feedback as the animal could interpret these trials as either being part of a two-trial or a one-trial reward block depending on his percept.

**Figure 2:**
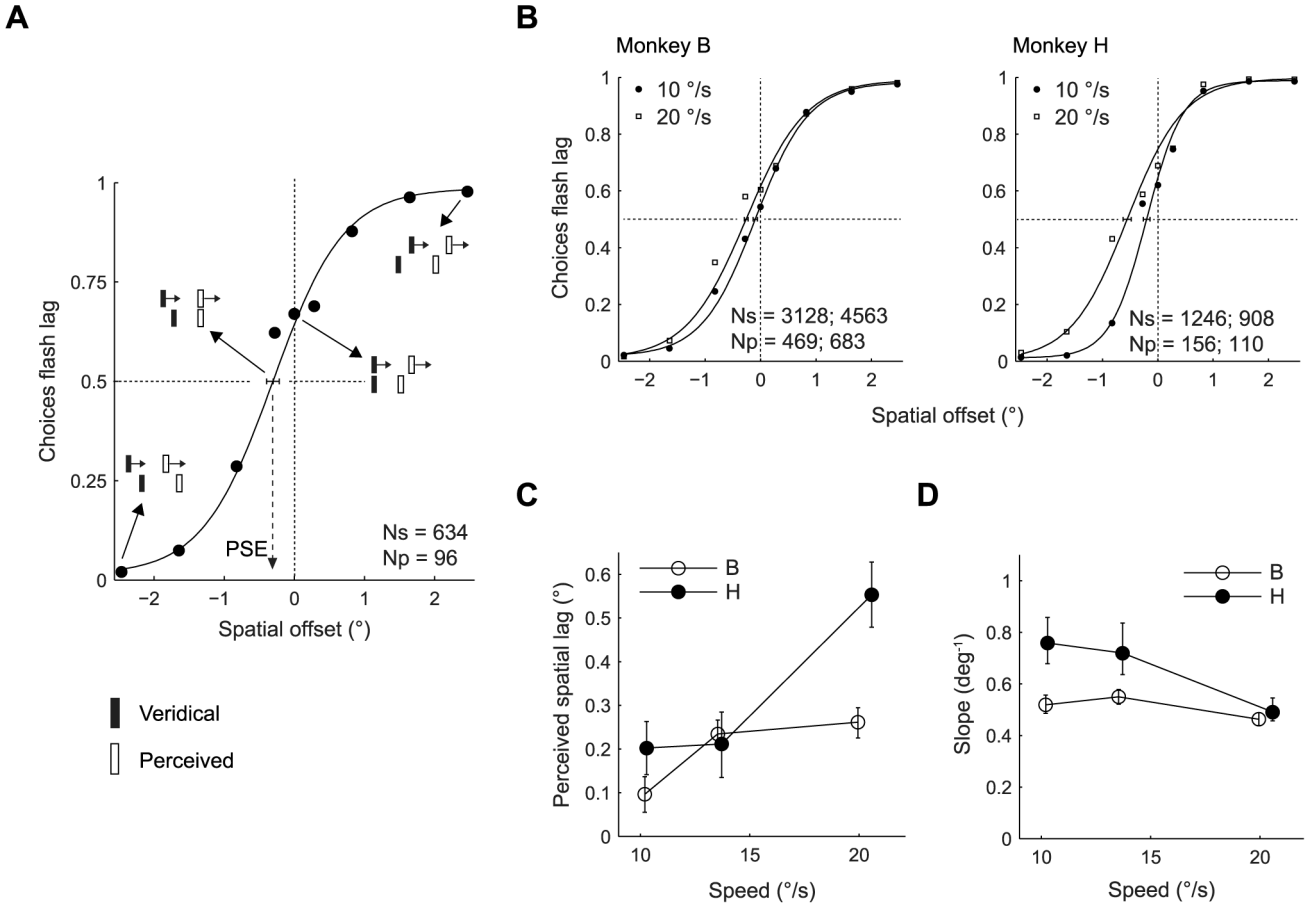
Monkeys perceive the flash lag illusion. (A) Psychometric function from a single session of monkey B for a moving bar speed of 20°/s. The abscissa shows the veridical spatial offsets of the stimuli. The ordinate shows the probability of the monkey reporting that the flash lagged behind the moving bar. Four different points along the psychometric function are illustrated with cartoons of veridical stimuli (filled vertical bars) and hypothetical perceived stimuli (open vertical bars). The point of subjective equality (PSE) is the spatial offset at which the monkey reports the flash as lagging in 50% of trials (i.e. he perceived the two bars as vertically aligned). In this session the PSE was −0.31°, indicating that the flash had to be placed 0.31° ahead of the moving bar to make them perceptually aligned. Ns: average number of trials per suprathreshold spatial offset data point (spatial offset magnitude >1.5°); Np: average number of trials per subthreshold spatial offset data point (spatial offset <1.5°). Error bar: 95% bootstrap percentile-based plug-in estimate of confidence interval. (B) Psychometric function fit of the pooled responses (5–8 sessions) of monkeys B and H. For clarity, data from only two speeds (10 and 20 °/s) are shown. Ns and Np: as in A. For the speed that is not shown (14 °/s): for monkey B, Ns = 4061 and Np = 628; for monkey H, Ns = 852 and Np = 108. Error bars as in A.(C) Perceived spatial lag for monkey B (open circles) and monkey H (filled circles), as a function of the speed of the moving bar. Each data point is the PSE estimated from the psychometric function fit (shown in panel B) of responses pooled from five to eight sessions. Error bars as in A.(D) Slope of the psychometric functions measured at the threshold point (0.5 on the ordinate). Coding and error bars as in C.

The behavioral responses of the monkeys indicate that they perceive the flash lag effect (Figure 2). The psychometric function for monkey B obtained in an example session is shifted significantly to the left: when flashed and moving bar were aligned (spatial offset: 0°) the monkey was more likely to report the flashed bar as lagging behind the moving bar (∼65% of trials) than the opposite (Figure 2A). If the monkey's percept was identical to the physical reality, the curve should go through 0.5 on the ordinate. Thus, the monkey perceived the flash as lagging behind the moving bar.

We quantified the perceived lag at multiple speeds (10, 14 and 20°/s) of the moving bar for both monkeys by fitting a psychometric function to the monkeys' responses (Figure 2B). From this fit, we estimated the veridical spatial offset at which the monkey chose left and right with equal probability, referred to as the point of subjective equality (PSE) or the perceived spatial lag. We found that the perceived spatial lag increased with the speed of the moving bar in the monkeys (Figure 2C; linear mixed model analysis was used for all statistical tests in this study; F(1, 24.6)  = 76.7, p<0.001), similar to what has been reported in humans [6], [26], [27].

While the point of subjective equality estimates the bias in the spatial localization of the moving bar, the slope of the psychometric function reflects the variability or uncertainty in judging the relative position of the moving bar; a steeper slope indicates a relatively lower localization uncertainty or higher stimulus discrimination sensitivity. Given that in humans the perceptual uncertainty in localizing a moving stimulus increases with speed [28], we wanted to see how the speed of the moving bar affects the slope of the psychometric function in monkeys (Figure 2D). Indeed, the slope decreased significantly as the speed increased (F (1, 26)  = 48.4; p<0.001), indicating that the monkeys' uncertainty in localizing the moving bar increased with the speed.

### Human psychophysics

**Figure 3:**
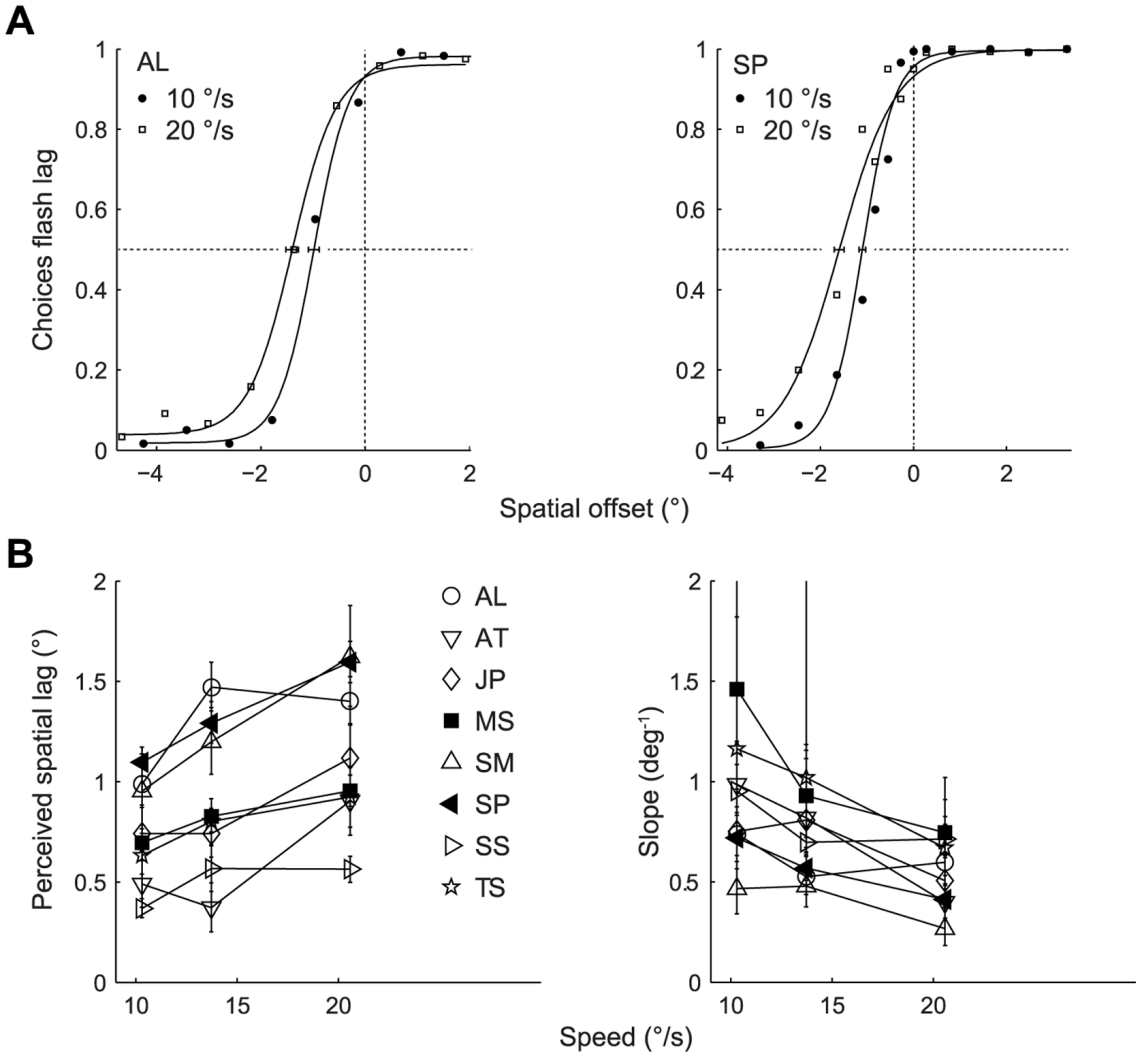
Flash lag illusion in humans. (A) Example psychometric functions from a naïve (left panel) and a non-naïve (right panel) human subject measured at two speeds of the moving bar. Each data point shown was computed from 120 (subject AL) or 126 (subject SP) trials pooled from three to four sessions. Error bars: 95% bootstrap percentile-based plug-in estimate of confidence intervals.(B) Perceived spatial lags (left panel) and slopes (right panel) of the psychometric function measured at different speeds of the moving bar from eight human subjects. Each data point represents the relevant parameter extracted from psychometric functions fitted (as in panel A) on responses pooled across two to five sessions. Open symbols: naïve subjects; filled symbols: non-naïve subjects. Error bars as in A. Slope error bar upper limits for subject TS are cropped: values are 6.5 and 6.1 for speeds 10 and 14 °/s respectively).

To compare the characteristics of the flash lag illusion in monkeys and humans we measured the flash lag effect in humans under identical stimulus conditions. As expected, the responses of human subjects indicated that they perceived the flash lagging significantly behind the moving bar (Figure 3A). We again varied the speed of the moving bar. As reported previously for similar stimuli in humans [27], the perceived lag increased linearly with speed (F (1, 47.9)  = 86.4; p<0.001) (Figure 3B, Left). In addition, the slope of the psychometric function (Figure 3B, Right) decreased significantly as a linear function of speed (F (1, 36.7)  = 13.4; p = 0.001) similar to the trend observed in the monkeys.

### Monkey versus human comparison

**Figure 4:**
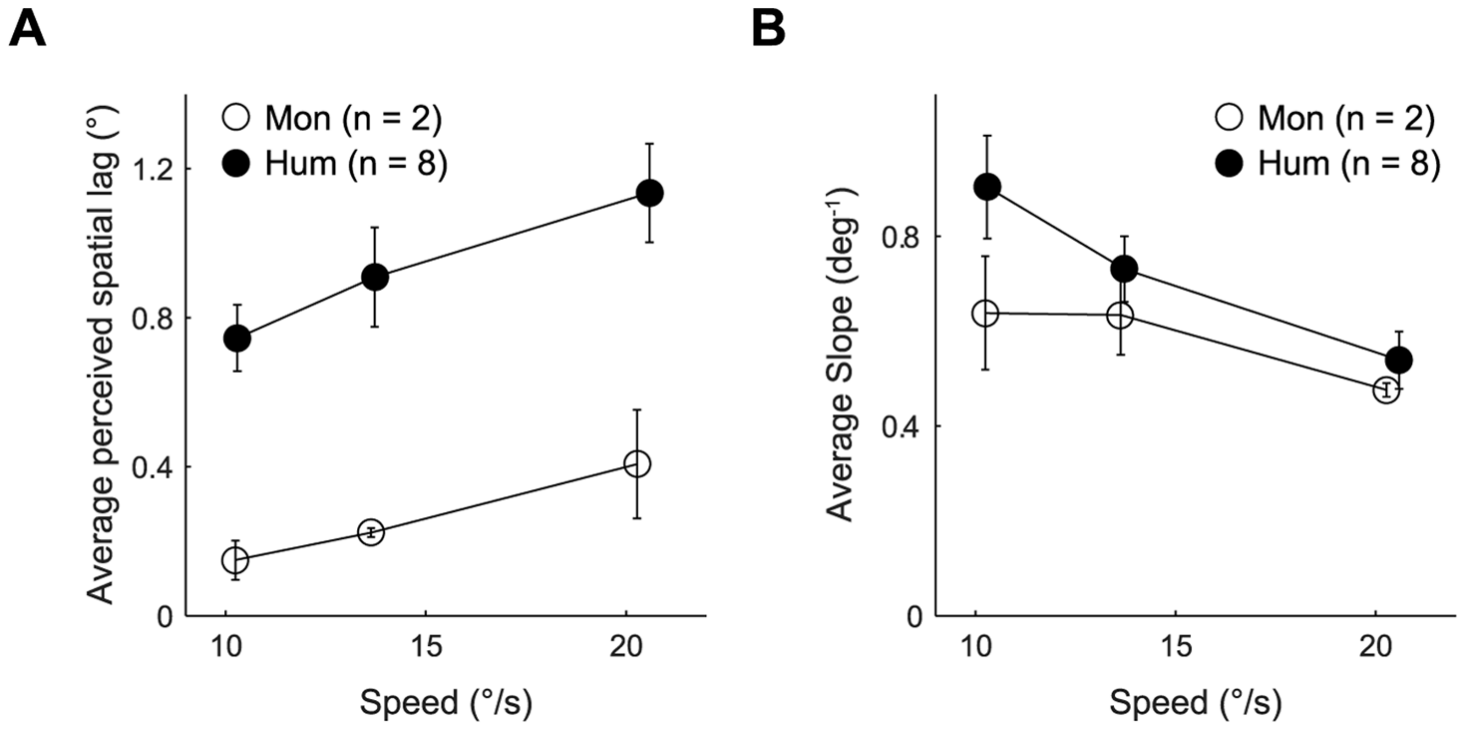
Comparison of illusion characteristics in monkeys and humans. (A) Perceived lag in monkeys (open circles) and humans (filled circles) averaged over subjects as a function of speed of the moving bar. Error bars: ±1SEM.(B) Slope of the psychometric function averaged over subjects. Coding and error bars as in A.

Next, we compared the flash lag illusion and its speed dependence in monkeys and humans (Figure 4). We found that the magnitude of the illusion is significantly lower in monkeys than in humans in the range of speeds we tested and that the perceived lag increased with speed in both species (significant main effects: species, F (1, 10.9)  = 17, p = 0.002; speed, F (1, 72.4)  = 139.6, p<0.001; non-significant species × speed interaction, F (1, 71.4)  = 2.6, p = 0.109). The results also indicated that macaques and humans are similar in stimulus discriminability, as the slopes were comparable in magnitude and decreased with speed in both species at the same rate (significant main effect of speed, F (1, 59.9)  = 22, p<0.001; non-significant main effect of species, F (1, 23.7)  = 2.7, p = 0.116; non-significant species × speed interaction, F (1, 59.5) = 1.1, p = 0.292). Thus the speed dependence of the flash lag illusion characteristics measured is similar in monkeys and humans, whereas the overall magnitude of the illusion is significantly smaller in monkeys than in humans.

**Figure 5:**
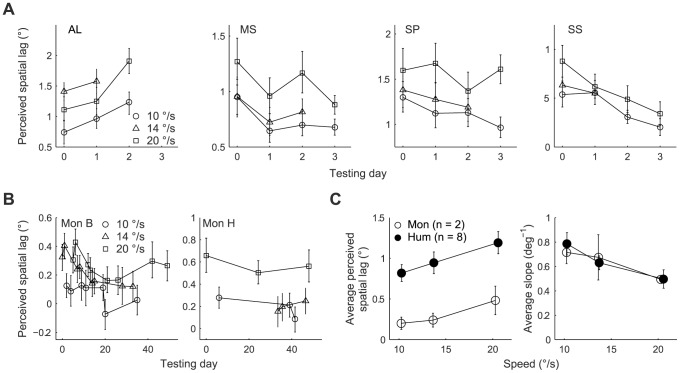
Change in perceived lag in monkeys and humans over time. Perceived lag measured at three speeds of the moving bar over the course of testing on multiple days in humans (panel A) and monkeys (panel B). (A) Each data point represents one to two sessions (sessions within a day were pooled). Subject AL and SS showed a significant increase and decrease, respectively, in perceived lag over testing days. Error bars: 95% bootstrap percentile-based plug-in estimates of confidence intervals.(B) Each data point represents a single session for monkey B and up to three consecutive non-overlapping sessions for monkey H (sessions were pooled if necessary, to meet a criterion of a minimum of five trials per stimulus condition). Error bars as in A.(C) Perceived lag (left panel) and slope (right panel) of psychometric function estimated from only the first one to three testing days in monkeys and humans and averaged over subjects. Error bars: ±1SEM.

Given that we used identical stimulus configurations for monkeys and humans, our finding that the lag was substantially smaller in monkeys was unexpected. We noticed that the perceived lag changed as a function of testing time in monkeys and humans (Figure 5). We therefore asked whether this change could have contributed to the differences between monkeys and humans.

In monkeys, the spatial lag decreased significantly with testing time without affecting its speed dependence (significant main effects: speed, F(1, 25.6)  = 77.9, p<0.001; testing day, F(1, 4.1)  = 12.1, p = .025; non-significant speed × testing day interaction, F(1, 24.4)  = 1.5, p = 0.238). In humans, we restricted our analysis to four of the eight subjects from whom we had collected data under the same stimulus conditions for at least three days. When the four human subjects were treated as a group, there was no significant change in the perceived lag over time (significant main effect speed, F(1, 30.2)  = 79.5, p<0.001; non-significant main effect of testing day, F(1, 28.8)  = 0.213, p = .648; non-significant speed × testing day interaction, F(1, 29.6)  = 1.9, p = 0.179). However, at the individual subject level, we noticed a declining trend in the perceived lag over testing day in three of the four subjects (Figure 5A) similar to what we had found in monkeys, although the effect was significant only in one of the three subjects (subject SS, significant main effects: speed, F(1, 6.9)  = 12.2, p = 0.01; testing day, F(1, 2.5)  = 69.5, p = 0.007; non-significant speed × testing day interaction, F(1, 4.4)  = 2, p = 0.227). In the fourth subject, we found a significant increase in the perceived lag over testing days (subject AL, significant main effects: speed, F(1, 4.4)  = 23, p = 0.007; testing day, F(1, 1.4)  = 55.4, p = 0.044; non-significant speed × testing day interaction, F(1, 3.2)  = 5.8, p = 0.091). Taken together, these data suggest that the perceived lag likely changes over testing time in the subjects of both species. Irrespective of the trend of the change in perceived lag over time, the main conclusions remain unchanged: if we used only sessions from first one to three testing days in monkeys and humans the perceived lag is smaller in monkeys, monkeys and humans show similar speed dependence of illusion characteristics, and both species have comparable stimulus discriminability in our task (Figure 5C) (perceived lag: significant main effects: speed, F(1, 15.9)  = 52.7, p<0.001; species, F(1, 6.9)  = 9.5, p = 0.018; non-significant species × speed interaction, F(1, 16.3)  = 0.508, p = 0.486; slope of the psychometric function: significant main effect of speed, F(1, 14.1)  = 20, p 0.001; non-significant main effect of species, F(1, 13.9)  = 0.0, p = 0.997; non-significant species × speed interaction, F(1, 20.7)  = 0.24, p = 0.627).

## Discussion

The present study shows that macaque monkeys perceive the flash lag illusion. Our reward paradigm allowed us to probe the illusion characteristics unbiased by reward contingencies. Using identical stimulus conditions, we established that the speed dependence of the illusion characteristics is similar in monkeys and humans. However, the magnitude of the illusion is significantly smaller in monkeys. Also, humans and monkeys showed similar stimulus discriminability as measured by the slope of the psychometric functions. Taken together, our data suggest that the flash lag illusion is qualitatively similar in the two species.

A fundamental difficulty in probing if non-human primates perceive an illusion is the reward. In most of the previous reports that studied illusions in macaque monkeys, the monkeys were explicitly rewarded, although randomly, independent of the animals' response direction for the illusion stimuli in probe trials [29]–[35]. Although such a reward strategy can work well in many cases, it can encourage monkeys to respond randomly on the probe trials in the long run, resulting in underestimating the true illusion magnitude or promoting idiosyncratic response strategies. We minimized these issues by first training the monkeys on suprathreshold trials to expect reward only after completing a small number of correct trials, before injecting probe trials that were not immediately rewarded. This initial training conditioned the monkeys to report their true percepts without receiving immediate reward. During the testing phase, we continued this conditioning using a large proportion (85%) of suprathreshold trials. Due to this conditioning, the monkeys were motivated to perform well and report their true percepts on the probe trials as well, which formed a small proportion (15%) of the total trials. A few studies have used a similar reward strategy, although the monkeys had to wait for a longer period of time before they received reward [36], [37]. One important caveat to consider regarding the reward paradigm implemented here is that over the period of extensive testing, the monkeys may learn that a sincere response is not necessary in the subthreshold/probe trials to receive reward. However to do so, the monkeys have to ‘tag’ the subthreshold trials and become ‘lazy’ on those specifically. First, given that the majority of the trials are suprathreshold trials, on which they had to perform well to receive reward, it is less likely that they would use a different response strategy for the remaining 15% of the trials. Second, the behavioral data suggest that the monkeys did not perform randomly in the subthreshold trials. For, if this were the case, the central five points in the psychometric function should lie close to 0.5; however, the psychometric function was smoothly ‘S’ shaped indicating that the monkeys did not perform badly in the subthreshold trials. Finally, the training paradigm is set up in a way that provides minimal feedback to the monkeys.

We considered any shift in the psychometric function towards negative spatial offsets as evidence for the monkeys perceiving the illusion. Could other factors than a true perceptual bias give rise to such a shift? We can rule out simple motor or oculomotor system response biases since in all our test sessions, we presented two motion directions with equal probability. The reward scheme could not have caused a shift either, since in probe trials no particular response was reinforced. All spatial offsets were equally likely positive or negative and the window of offsets that were considered subthreshold was symmetric about zero. Thus, the data strongly suggest that the shift in the psychometric function reflects the monkeys' perceptual bias arising from the flash lag illusion.

The illusion magnitude observed in monkey H was higher in general compared to that in monkey B. Here we consider some of the likely contributing factors. Note that monkey H could respond with the joystick any time after the moving bar started to move whereas monkey B had to wait 200 ms after the flash was presented before making a saccadic response. In addition, the contrast of the bars was lower for monkey H compared to that used for monkey B. Hence, differences in stimulus contrast, method of responding (oculomotor versus skeletomotor) and response wait time could all have contributed in principle to the difference in the illusion magnitude between the two monkeys. However, it is worth noting that even in human subjects, where identical stimulus presentation conditions were used for all observers, the illusion magnitude was variable among observers similar to what we observed in monkeys. This would suggest that individual differences could be a significant contributor to the difference in the illusion magnitude observed in the two monkeys.

In both monkeys and humans in our study the perceived lag increased linearly with speed (Figure 4A) and the slope of the psychometric function decreased linearly with speed (Figure 4B) suggesting that the spatio-temporal localization mechanisms as revealed by the flash lag illusion are likely to be similar in both species. However, the magnitude of the flash lag illusion was substantially lower in monkeys than in humans. This was striking given that all other characteristics of the illusion tested were similar in the two species. One factor that could in principle have contributed to this difference is eye movements: while the monkeys were required to fixate within a window of 1–1.5° in diameter, the humans were only instructed to fixate and therefore may have fixated in a larger window or possibly tracked the moving stimuli. Although we cannot rule out this possibility with our data, it seems unlikely to play a major role since tracking the moving bar has been reported to decrease the illusion magnitude [38] – which would make the opposite prediction. In the monkeys, the narrow fixation window rules out eye movements as a factor in underestimating the true illusion magnitude.

A second factor that could have contributed to the difference between humans and monkeys is training: the monkeys were extensively trained for several weeks on suprathreshold stimuli before they were shown illusion stimuli, whereas humans were trained only for a few minutes on the suprathreshold stimuli before testing. The species difference of the illusion magnitude remained significant even if we used only the first few testing sessions, ruling out the possibility that it was caused by pooling data collected over a longer period of time in monkeys. However, the extensive initial training using suprathreshold stimuli, is an unavoidable confound that could have contributed to the lower illusion magnitude in monkeys compared with humans. New behavioral training paradigms with shorter training time could help to alleviate this issue in future studies. In addition, our boundary between sub- and suprathreshold trials may have been somewhat too small at 1.5°, which is close to the PSE for some of our human subjects (it was picked based on a pilot study with fewer human subjects who had smaller PSEs). As a result, we may have rewarded the monkeys for reporting the veridical stimulus configuration rather than reporting their true percepts for spatial offsets with magnitude around 1.5° and higher; this could have shifted the true psychometric function to the right, thereby underestimating the illusion magnitude.

Our finding that the perceived spatial lag decreased with time in the monkeys was unexpected. One factor that has been shown to affect the magnitude of the flash lag illusion in human subjects is attention [39]. Hence, one possible explanation is that, since we presented the flashes at two fixed locations, the monkeys' spatial attention to the flash locations may have improved over time, explaining the decrease in the perceived lag. Although in monkey B there is a clear decreasing trend in the illusion magnitude over time, the trend is not as pronounced in monkey H and the trend is not uniform among the four human subjects, indicating that more data will be necessary to make a strong conclusion on the change in the illusion magnitude over time. Nonetheless, further modifications in the current behavioral paradigm or new behavioral paradigms, with shorter training and testing time and recording from large number of neurons simultaneously (using multi-electrodes arrays for example) could help to alleviate the potential issue of changes in illusion magnitude over time in future physiological studies of this fascinating illusion.

### Conclusions

Our study demonstrates that macaque monkeys perceive the flash lag illusion. Many of the illusion characteristics were comparable in monkeys and humans. However, the magnitude of the perceived illusion was much lower in monkeys than in humans, emphasizing the point that species differences should be characterized and taken into account when extending results from animal models to humans. Our observations that the illusion magnitude changes over time underscores the importance of taking the time factor into consideration in designing experiments and interpreting results. The flash lag illusion, although originally observed in the visual system with moving stimuli, has now been demonstrated in other modalities [40]–[42] and in more general settings where stimulus dimensions such as color, luminance, spatial frequency and pattern entropy change continuously [14]. The widespread presence of this phenomenon in the brain underscores the generality of the underlying neural mechanisms. The methods developed here to train monkeys to report their true percepts and the behavioral results can be used as a guide in designing future animal model studies that combine neurophysiological and behavioral analysis to understand how our brain represents time-varying signals accurately in different modalities.

## Methods

### Ethics statement

The surgical and experimental procedures on monkeys were approved by the Institutional Animal Care and Use Committee (permit number: AN-4367) of Baylor College of Medicine. The animals were housed individually in a large room located adjacent to the training facility, along with around twenty other monkeys permitting rich visual, olfactory and auditory interactions. Regular veterinary care and monitoring, balanced nutrition and environmental enrichment were provided by the Center for Comparative Medicine of Baylor College of Medicine. Surgical procedures on monkeys were conducted under general anesthesia following standard aseptic techniques. To ameliorate pain after surgery, analgesics were given for 7 days. Animals were not sacrificed after the experiments. Experimental procedures with human subjects were approved by the Institutional Review Board (permit number: H-21874) of Baylor College of Medicine. Written informed consent was obtained from human subjects. For completion of experimental sessions, monetary compensation was given to the naïve human subjects.

### Subjects

Two male macaque (*Macaca mulatta*) monkeys (B and H) weighing 11 and 17 kg, aged 11 and 10 years respectively, and eight human observers (23–49 years old; six naïve and two non-naïve (SP and one of the authors -MS)) participated in the experiments. All human subjects had normal or corrected to normal vision.

### Visual stimulus presentation

Visual stimuli were presented on CRT monitors (model: Sgi C220 Flat Diamondtron; display size: 22°×16° from a distance of 100 cm; resolution: 1600×1200 pixels; refresh rate: 100 Hz) from Macintosh computers using Psychophysics Toolbox 3 [43]–[45] in a dark room. The monitors were gamma corrected to have a linear luminance response profile. The monitor background luminance was 8 cd/m^2^ (monkey H) or 10 cd/m^2^ (monkey B and all human subjects). Moving and static vertical bars of identical luminance and size (0.3°×4.4°) were used as visual stimuli. For monkey B and for all human subjects, the bars had a luminance of 44 cd/m^2^; for monkey H, in a given session one of four bar luminances (10, 16, 21 and 25 cd/m^2^) was used (we report results only for the bar luminance of 25 cd/m^2^). During the stimulus period (Figure 1B and Behavioral Task section), the moving bar started translating horizontally from one end of the monitor screen; at a specific time of motion, the static bar was flashed below the moving bar trajectory for one video frame (10 ms) at a given location. The onset time of the flashed bar relative to the onset time of the moving bar was varied to create different horizontal spatial offsets between the moving and flashed bar centers; the vertical offset (0.8°) between the bottom edge of the moving bar and the top edge of the flashed bar remained constant. In the first two phases of monkey training (see Behavioral Task section), the horizontal offset magnitude was fixed (2.7° to 3.2°) for a given session and the flash location was randomly chosen on every trial. During the final training phase, a range of horizontal offsets (suprathreshold offsets: ±2.5°, ±1.6°; subthreshold offsets: ±0.82°, ±0.28°, 0°; positive sign indicates that the flash was presented behind the moving bar) were used with the flash location centered at one of two fixed locations symmetric around the vertical meridian in the lower visual field at an eccentricity of 3.9° (azimuth: ±1.4°, elevation: 3.6° below horizontal meridian). For subject H, two additional flash locations (7.2° eccentricity, azimuth ±6.2°) were used. The moving bar translated at 10, 14, 20 °/sec from left to right or from right to left in the upper visual field following a trajectory length of 22° or 24°. The trajectory center was located along the vertical meridian at an eccentricity of 1.4°.

### Behavioral task – monkeys

The monkeys sat in a custom primate chair at a distance of 100 cm (monkey B) or 107 cm (monkey H) from the stimulus display, with their heads restrained. Cranial head post and scleral eye coil were implanted under general anesthesia using aseptic techniques. For monitoring eye movements, scleral search coil was implanted in monkey H; for monkey B, a custom video eye tracking software developed in LabView (camera: DALSA Genie HM640, frame rate: 200 Hz) was used. For both monkeys, eye movement traces were sampled at 2 KHz. A trial (Figure 1B) began with a brief sound. The monkeys were required to start fixating their gaze at a red fixation spot (0.14° diameter) within a circular window of 1° (monkey H) or 1.5° (monkey B) diameter at the monitor center within 4 seconds of the start of the trial. After the monkeys maintained fixation for 300 ms, the stimulus period began. A two alternative forced choice paradigm was used to train the monkeys to report if the flashed bar was located on the left side or right side of the moving bar. To report their choice, monkey H moved a lever either left or right and monkey B saccaded to one of two saccade targets (circular white patches of 0.8° diameter, located in the upper visual field, outside the moving bar trajectory). For monkey H, the response period began 200 ms after the moving stimulus onset; for monkey B, to prevent premature responses, the response period began 200 ms after the flash offset; for both monkeys, the response period ended 4500 ms after termination of the moving bar presentation. Auditory feedback about the monkeys' choice was given only during the first two phases of training (see below). The fluid reward schedule for completing the trials is described below. The next trial began after an inter-trial period of 1200 ms.

### Training paradigm

The goal of the behavioral training was to measure the magnitude of the flash lag illusion unbiased by reward contingencies using the method of constant stimuli. Typically, in operant conditioning, the experimenter defines what response is correct and trains the animals to respond accordingly by reinforcing correct responses with reward. When ‘correct’ responses are not well defined (as is the case for illusions), one strategy would be to use a random reward scheme for the probe/subthreshold trials. However, under this scheme, as the response is explicitly dissociated from reward, the monkey could be encouraged to respond randomly on the probe trials. Consequently, this could lead to a negative result or underestimation of the true size of the effect that one seeks to measure. We attempted to minimize this potential problem by a reward scheme where the monkeys never get immediate reward for the probe trials, thereby eliminating explicit dissociation of reward and responses. However, at the same time, we designed the task in such a way that the monkeys are conditioned to believe that the probe trials are actually counted for reward. The detailed implementation of this idea is as follows.

The training was done in three phases. In the first phase, the moving and flashed bars were presented with suprathreshold offsets and every correct trial was immediately rewarded. The magnitude of the horizontal offsets for this step was determined from preliminary human psychophysical studies, where we observed that with spatial offsets greater than 1.5° in magnitude, the subjects' report matched the veridical stimulus configuration. With the assumption that the same held true for the monkeys, we trained them to report the relative location of the flash and rewarded them if their reports matched the veridical stimulus condition. In each session, the speed and magnitude of horizontal offset were fixed; the two motion directions were randomly interleaved; the flash location was randomly chosen every trial to discourage the monkeys from basing their decision on the moving bar location alone. Once the monkeys' reports matched the veridical stimulus conditions in more than 85% of the trials, they moved to the second phase.

The main goal of the second training phase (Table 1) was to train the monkeys to do the same task as in phase-I but without immediate reward for some trials (no-reward trials) while receiving immediate reward for the rest of the trials (reward trials). To achieve this, we rewarded the monkeys after completing a total of either one or two correct trials (one-trial and two-trial reward blocks respectively). The monkeys were allowed to attempt as many trials as needed to get the required number of total correct trials; for example, to get two correct trials, the monkeys can complete two correct trials contiguously in a row or can attempt five trials in which the first and last trial are correct and the rest are incorrect; in either case, the monkey gets reward after the last correct trial; hence the last trial is the reward trial and all preceding trials are no-reward trials. For every trial, a randomly chosen stimulus condition was presented. Once the monkeys adapted to this reward contingency and the performance stayed above 85% correct, they moved to the final phase of the training.

In the final phase (Table 1) we followed the same reward strategy as in phase-II. However, we interleaved subthreshold offsets with suprathreshold offsets. For the suprathreshold offset conditions, a trial was considered correct if the monkeys' report matched the veridical stimulus condition. For the subthreshold offset conditions, the monkeys' responses were always counted as correct. All the subthreshold offset conditions were presented only in the no-reward trials (first trial of the two-trial reward blocks). Hence the monkeys' choices were never biased by reward. The suprathreshold offsets were presented in the no-reward trials as well as in reward trials (under one-trial and two-trial reward blocks). The reward blocks were randomly interleaved with a proportion of 1∶1∶1 (one-trial reward block with suprathreshold offsets, two-trial reward block with suprathreshold offsets, two-trial reward block with sub- and suprathreshold offsets). Subthreshold and suprathreshold offsets were chosen from the respective set of offsets randomly. On a typical day, the monkeys performed an average of roughly 2000 trials, 15% of which were subthreshold offset trials, giving around 15 repetitions for each of the 20 subthreshold stimulus conditions (5 offsets ×2 directions ×2 flash locations ×1 speed ×1 bar luminance) for monkey B or around 7 repetitions for each of the 40 subthreshold stimulus conditions (5 offsets ×2 directions ×4 flash locations ×1 speed x 1 bar luminance) for monkey H.

### Behavioral task – humans

The stimulus and task details are the same as for the monkeys with the following exceptions. The human subjects sat in a comfortable chair and used a chin rest to minimize head movement. They were instructed to maintain fixation but their eye movements were not monitored. Subjects were instructed to respond with a lever or a keyboard as soon as the flash was presented; no feedback was given for responses. The inter-trial period was 500 ms. A short (∼5 min) session with large spatial offsets with auditory response feedback was used to train the naïve subjects. For measuring the illusion magnitude, the range of horizontal offsets was adjusted for each subject and for each speed based on the psychometric function obtained from the first session of each subject. In a single sitting, each stimulus condition was repeated 10 times in a block design. Most sittings consisted of 360 trials (9 offsets ×2 directions ×2 flash locations ×1 speed ×10 blocks) and lasted for 15–25 min. Three speeds were tested: 10, 14 and 20°/s, with two to five (mean: 3, SD: 1) sittings per speed.

### Data analysis

Data analysis was done in MATLAB using custom-written code. We fitted psychometric functions to the subjects' probability of reporting that the moving bar was located ahead of the flashed bar at different veridical spatial offsets, using the psignifit3 toolbox [46]–[48]. For each subject, we extracted the point of subjective equality (PSE; the veridical spatial offset at which subjects responded that the moving bar was ahead or behind the flashed bar with equal probability) for each speed separately, after pooling trials of all movement directions and flash locations. In the statistical tests addressing the trend in the perceived lag as a function of time (testing day), responses collected within the same day were first pooled before estimating the parameters of the psychometric functions. For all other statistical tests, the parameters were estimated for each session/sitting separately. For monkey H, the conditions where flashes were presented at an eccentricity of 7.2° were excluded from analysis since in those conditions the monkey's performance did not reach the criterion level of 90% correct for the suprathreshold offsets. To keep the stimulus conditions comparable between the two monkeys, we analyzed only one (25 cd/m^2^) of the four bar luminance conditions (10, 16, 21 and 25 cd/m^2^) from monkey H that was closest to that used in monkey B (44 cd/m^2^).

Statistical tests were done using the statistics software PASW 18. Linear mixed models were constructed with the following general settings: species of subjects, speed and time were treated as fixed effects; subjects were treated as random effects; perceived lag and slope of the psychometric functions were treated as dependent variables; speed of the moving bar and time were treated as continuous variables (covariates); repeated observations were identified by session start time or testing day with AR1 (first order autoregressive) as the covariance structure for the residuals. When an interaction term was not significant, it was removed from the model to reduce the model complexity and the model fitting routine was repeated before obtaining the significance levels for the main effects; hence, unless the interaction term was significant, the reported main effect significance levels were based on models without interaction terms.

### Data accessibility and reproducibility

All data and MATLAB code necessary to reproduce the results and figures presented in this paper are available online: http://dx.doi.org/10.5061/dryad.t1n04
