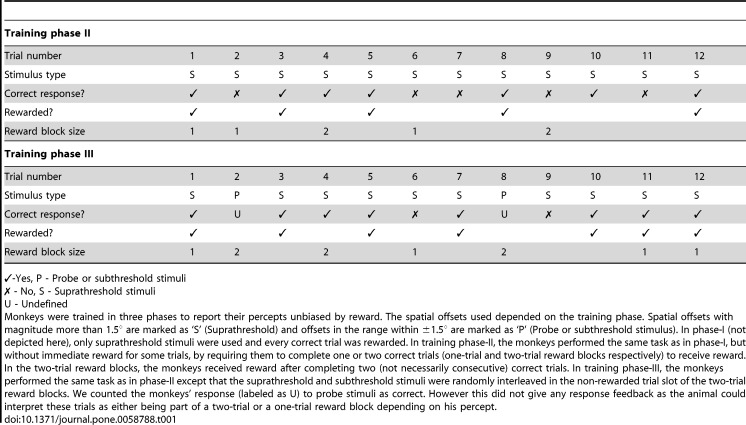# Correction: Macaque Monkeys Perceive the Flash Lag Illusion

**DOI:** 10.1371/annotation/2253d19d-1afb-4296-a4d2-ac84f1d808d0

**Published:** 2013-04-10

**Authors:** Manivannan Subramaniyan, Alexander S. Ecker, Philipp Berens, Andreas S. Tolias

In Table 1, in the second column of the "Correct response?" row of "Training Phase II", there was a missing "x" symbol. The correct Table 2 can be viewed here: 

**Figure:**